# The Roles of Insulin-Like Growth Factor 2 mRNA-Binding Protein 2 in Cancer and Cancer Stem Cells

**DOI:** 10.1155/2018/4217259

**Published:** 2018-03-15

**Authors:** Junguo Cao, Qingchun Mu, Haiyan Huang

**Affiliations:** ^1^Department of Neurosurgery, The First Hospital of Jilin University, Xinmin St. No. 71, Changchun, China; ^2^Hongqi Hospital Affiliated to Mudanjiang Medical University, Mudanjiang, China

## Abstract

RNA-binding proteins (RBPs) mediate the localization, stability, and translation of the target transcripts and fine-tune the physiological functions of the proteins encoded. The insulin-like growth factor (IGF) 2 mRNA-binding protein (IGF2BP, IMP) family comprises three RBPs, IGF2BP1, IGF2BP2, and IGF2BP3, capable of associating with IGF2 and other transcripts and mediating their processing. IGF2BP2 represents the least understood member of this family of RBPs; however, it has been reported to participate in a wide range of physiological processes, such as embryonic development, neuronal differentiation, and metabolism. Its dysregulation is associated with insulin resistance, diabetes, and carcinogenesis and may potentially be a powerful biomarker and candidate target for relevant diseases. This review summarizes the structural features, regulation, and functions of IGF2BP2 and their association with cancer and cancer stem cells.

## 1. Introduction

Insulin-like growth factor (IGF) 2 is a member of the insulin family of polypeptide growth factors which regulate development and growth [1, 2]. Its expression is delicately controlled during development through epigenetic, transcriptional, and translational mechanisms [2, 3]. It is also modulated posttranslationally by RNA-binding proteins (RBPs) which bind to IGF2 transcripts and mediate their processing, such as localization, stability, and translation [4, 5]. These include three *IGF2* mRNA-binding proteins (IGF2BPs, IMPs), IGF2BP1, IGF2BP2, and IGF2BP3, which interact with the leader 3 mRNA 5′ untranslated region of *IGF2* [6–8]. IGF2BPs share highly identical structural features with two N-terminal RNA recognition motifs (RRMs) and four C-terminal heterogeneous nuclear ribonucleoprotein K-homology (KH) domains [9], which confer IGF2BPs the affinity towards RNA and the regulation capacity of multiple target transcripts [4].

IGF2BP2 was originally identified as an RBP capable of binding *IGF2* mRNA [9], and later studies suggest that it also targets other transcripts, such as *LAMB2* [10], *LIMS2* [11], *TRIM54* [11], *UCP1* [12], and 12 other genes encoding mitochondrial components [12]. This multitargeting feature correlates with the wide range of physiopathological functions of IGF2BP2 in embryonic development, neuronal differentiation, lipid metabolism, insulin resistance, and tumorigenesis [13, 14]. High expression of IGF2BP2 during embryonic development coincides with its inhibitory role in mouse neocortex precursor cells' differentiation [15, 16] as well as energy metabolism, likely through its binding to the mRNAs that encode proteins forming mitochondrial respiratory complexes [17]. Recent genome-wide studies suggest *IGF2BP2* as a susceptibility gene for human type 2 diabetes mellitus (T2DM), and a single nucleotide polymorphism (SNP, rs4402960) in the second intron is associated with T2DM [18–20].

Accumulating evidence links IGF2BP2 with cancer. The T2DM-relevant SNP rs4402960 in *IGF2BP2* gene increases the risk of breast cancer in at least some ethnic groups [21]. Autoimmune response to IGF2BP2 observed in hepatocellular carcinoma (HCC) and colorectal, ovarian, and breast cancer supports the potential of the autoantibody against IGF2BP2 as a biomarker in cancer screening, diagnosis, and prognosis [22–24]. Indeed, overexpression of IGF2BP2 in basal-like breast cancer and esophageal adenocarcinoma predicts short survival of the patients [25–27]. At the cellular level, IGF2BP2 enhances genomic instability [27] and stimulates cancer cell proliferation and migration [28, 29]. Furthermore, studies from independent laboratories suggest that IGF2BP2 participates in the maintenance of cancer stem cells (CSCs) [17, 27, 30], implicating that IGF2BP2 may be important for the chemoresistance and recurrence of the diseases.

Despite the consolidated evidence for the physiopathological significance of IGF2BP2, our knowledge of its functions in embryonic development, energy metabolism, and disease progression is incomplete. Our understanding of IGF2BP2 functions at the molecular level is limited to glucose metabolism [19], mitochondrial activity [12, 17], and energy preservation [12]. In the past few years, more experimental evidence has linked IGF2BP2 to the progression of cancer, including HCC, glioblastoma, and breast, ovarian, colon, and esophageal cancer, particularly the maintenance of cancer stem cells. This review aims to summarize these advances along with the origin, structure, regulation, and functions of IGF2BP2. IGF2BP1 and IGF2BP3 are partially covered, in comparison with IGF2BP2 when necessary, to obtain a panoramic view of IGF2BP2 expression and function, particularly but not exclusively, in disease control and progression.

## 2. Origin of IGF2BP2

**Figure 1:**
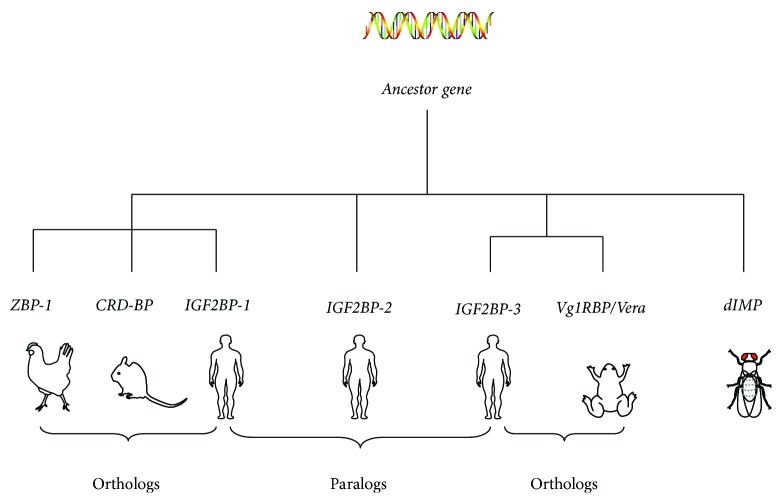
Phylogenetic analysis of genes encoding IGF binding proteins in *Homo sapiens*, *Mus musculus*, *Gallus gallus*, *Xenopus laevis*, and *Drosophila melanogaster*, using MacVector ClustalW alignment program.

Homologs of IGF2BPs have been identified in both invertebrates and vertebrates, although they are reported with different nomenclatures and their target mRNAs and biological functions may vary. These include human IGF2BPs, murine coding region determinant-binding key protein (CRD-BP), chicken zipcode-binding protein (ZBP1), and *Xenopus laevis* Vg1RBP/Vera. Phylogenetic analysis suggests that *IGF2BP1*, *IGF2BP2*, and *IGF2BP3* homologs in vertebrates and *Drosophila dIMP* originated from a common ancestor (Figure 1) [31]. Human *IGF2BP1*, mouse *CRD-BP*, and chicken *ZBP-1* are almost identical [32]. Human *IGF2BP3* and *Xenopus laevis Vg1RBP/Vera* are orthologs [31, 33, 34]. Homologs of human *IGF2BP2* have been identified as *IGF2BP2a* and *IGF2BP2b* in *Danio rerio* [31]. These IGF2BP homologs all have multiple conserved RNA-binding domains and are believed to mediate mRNA localization and translation through conserved mechanisms [31].

## 3. Gene and Protein Structure of IGF2BP2

**Figure 2:**
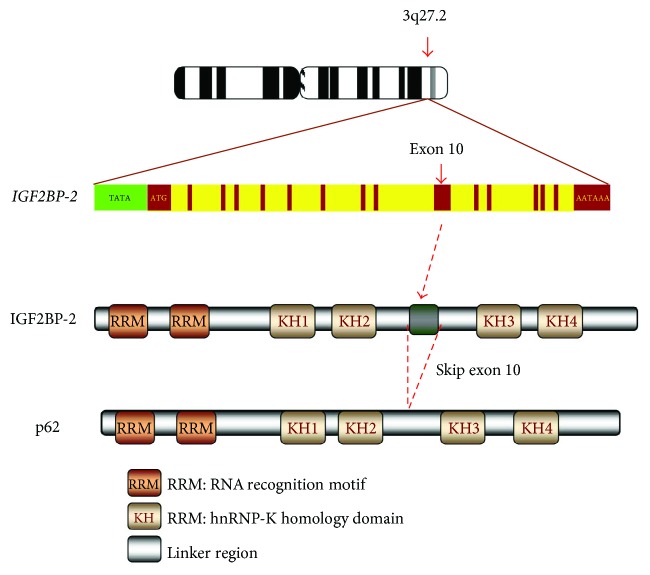
The gene and protein structure of IGF2BP2. Gene *IGF2BP2* contains 16 exons at chromosome locus 3q27.2. The IGF2BP2 protein has six RNA-binding domains, including two N-terminal RRMs and four C-terminal KH domains. The p62 isoform has a 43-aa loss between the KH2 and KH3 domains due to the skipping of exon 10.

Gene *IGF2BP2* contains 16 exons at chromosome 3 locus 3q27.2 [19], encoding a 66 kDa full-length protein [9]. Alternatively, it generates a splicing variant by skipping exon 10 and encodes a 62 kDa IGF2BP2 isoform, p62 (Figure 2) [35], which was identified as a cytoplasm-localized *IGF2* mRNA-binding protein and termed as IGF2BP2 [36]. IGF2BP2 shares with other IGF2BPs an overall 59% identity in amino acid (aa) sequence and a similar protein structure characterized by six RNA-binding domains, which includes two N-terminal RRMs and four C-terminal KH domains (Figure 2) [19]. p62 has a 43-aa loss between the KH2 and KH3 domains due to the skipping of exon 10 [35]. The six RBDs are highly conserved but spaced with linker regions which are more flexible in sequence and structure and determine the functional diversity of IGF2BPs. The RRM and KH domains commonly exist in numerous RBPs, such as heterogeneous nuclear ribonucleoproteins [37, 38], poly(A) RNA binding proteins, and the Vigilin family of proteins [4]; however, the tandemly arranged pattern of two RRM and four KH domains is unique to IGF2BPs [39]. The RRMs have high affinity for RNA, determining the binding capacities of the RBPs to RNA [40]. These motifs play a central role in the stability of IGF2BP-RNA complexes and coordinate the interactions between the complex and other RBPs [4, 40]. The KH domains primarily bind to RNA, preferentially the polypyrimidine region [41], although their association with DNA has also been demonstrated in *Xenopus laevis* [42], and the completion of KH domains in IGF2BP2 has been shown to be critical for its RNA-binding [43, 44].

Tandem repeats of RRMs and KHs in IGF2BPs are pivotal for their affinity, specificity, and versatility in RNA binding [19]. The contributions of individual RBDs are unclear and challenging to estimate, since RBDs, which share a high level of sequence identity, cooperatively engage in RNA binding. Repeated KH elements are important for high-affinity and specific RNA binding [40, 45]. RBDs also mediate the recruitment of other RBPs to the protein-RNA complexes [46, 47]. IGF2BPs form homologous and heterologous dimers in the presence of target mRNA and enhance stability of the protein-RNA complexes, adding another dimension of structural complexity and binding capacity, specificity, and versatility [48].

The linkers between RBDs of IGF2BPs are very important but easily overlooked. Phosphorylation of residues in these spacer sequences is key to the posttranscriptional control of the target mRNAs in cytoplasm. For instance, the linker between RRM2 and KH1 domains of IGF2BP2 can be activated by mTOR and promotes its binding to *IGF2* mRNA [49, 50]. Phosphorylation of the linker residues between KHs of ZBP-1 and V1RBP by Src and mitogen-activated protein kinase (MAPK) is essential for their association with *ACTB* mRNA, which is a prerequisite for the translocation and translation of *ACTB* mRNA in the cytoplasm during neuronal growth cone guidance [51–53].

## 4. Functions of IGF2BP2

### 4.1. IGF2BP2 in RNA Processing

**Figure 3:**
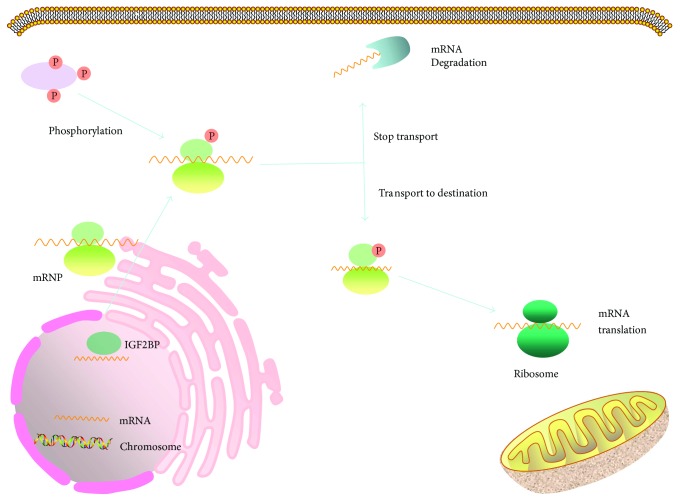
IGF2BPs regulate mRNA processing. IGF2BPs in the nucleus bind to the nascent mRNAs and join the assembly of messenger ribonucleoprotein (mRNP) complexes before exporting them to the cytoplasm. The mRNAs are then transported, in the form of mRNPs, along microtubules or other cytoskeletal structures to their destination in subcellular compartments. IGF2BPs maintain the stability of mRNAs and silence their translation during trafficking.

Our knowledge about the functions of IGF2BPs in RNA processing mainly comes from the studies on IGF2BP1, the best understood member of this RBP family. Briefly, IGF2BPs predominantly localize in the cytoplasm, where they form ribonucleoprotein particles (RNPs) with their target mRNAs [54, 55], but are also capable of entering the nuclei (Figure 3) [54], where they bind to nascent transcripts [56, 57] and attend their regulation [51, 55, 58]. Controlled by the nuclear export signals in the KH domains, IGF2BPs can transport the target transcripts out of the nucleus [54], protecting them from degradation [39, 59], and mediate their translation spatially and temporally [48, 50, 51, 60–62].

The function of IGF2BP2 in RNA processing is less understood compared with IGF2BP1; however, a number of client mRNAs for IGF2BP2 have been identified (Table 1). These include *IGF2* [49], *LAMB2* [10], *LIMS2* [11], *TRIM54* [11], and hundreds of transcripts immunoprecipitated with IGF2BP2 ribonucleoprotein complexes from gliomaspheres formed by glioblastoma stem cells [17]. *IGF2BP2* deletion in mice alters the translation of 15 IGF2BP2 target mRNAs, including 13 transcripts encoding mitochondrial components such as UCP1 [12]. In glioblastoma cells, IGF2BP2 facilitates the trafficking of mRNAs to the vicinity of mitochondria for subsequent translation and cellular functions [17]. Moreover, in a screening for RBPs that inhibited the AUF1-mediated degradation of mRNA using a yeast two-hybrid system, IGF2BP2 was identified to bind to AUF1 and prevent mRNA degradation, suggesting that IGF2BP2 functions as an mRNA stabilizer [63]. In addition, phosphorylation of IGF2BP2 in the linker region between RRM2 and KH1 by mTOR promotes its binding to the IGF leader 3 mRNA 5′-UTR, enhancing the initiation of IGF2 translation through eIF-4E- and 5′ cap-independent internal ribosomal entry [49]. These findings suggest that IGF2BP2 has multiple functions in RNA processing like IGF2BP1.

### 4.2. Physiological Activities of IGF2BP2

#### 4.2.1. Embryo Development

A biphasic expression pattern during early embryogenesis and later developmental stages is commonly observed for IGF2BPs in different species. *IGF2BP1*, *IGF2BP2*, and *IGF2BP3* mRNAs were detected at the two-cell stage during mouse embryogenesis and undetectable thereafter until E11.5 days, and the expression was then maintained till birth [9, 64]. Similar expression patterns have been observed in *Xenopus laevis*, *Danio rerio*, and *Drosophila melanogaster* [35, 65–67].

In spite of the similarity in the timing of expression, variations have been found in their spatial distribution. IGF2BP1 and IGF2BP3 are mainly expressed in the forebrain, afterbrain, snout, branchial arch, viscera, skin, and tail vertebrae [66, 68, 69], whereas IGF2BP2 is usually present in brain tissue (including cerebral cortex, striatum, and ventricle), nasal cavity, lung, liver, intestine, kidney, and other tissues [19]. Towards the end of embryonic development, IGF2BP3 gradually recedes, and a residual level of IGF2BP1 remains in the small and large intestine, kidney, and liver [68–70]. In contrast, IGF2BP2 expression is continued in brain, viscera, bone marrow, kidney, lung, muscle, liver, testis, and pancreas [71, 72]. The tissue-specific expression of IGF2BPs highly overlaps with that of IGF2 [9], consistent with the regulatory hierarchy. The temporal and spatial expression of IGF2BP1, IGF2BP2, and IGF2BP3 supports their essential roles in embryonic development. In accord with this, dwarfism and impaired gut development have been demonstrated in *IGF2BP1*-deficient mice [68]. Loss of *dIMP* function in *Drosophila* is zygotic lethal while gain of function disrupts dorsal/ventral polarity [39, 73]. *IGF2BP2*-null mice are lean and gain less weight than their wild-type littermates [12]. In addition, a wide range of expression of IGF2BP2 in adult tissues, particularly gut, muscle, and brain, suggests that this protein may also regulate other physiological processes, such as food uptake, feeding behavior, and metabolism as previously reviewed [19].

#### 4.2.2. Nervous System

Expression of mouse IGF2BP2 during the early embryonic development and its decrease after birth [74] correlate with its regulatory role in the differentiation of neural precursor cells into neurons or glial cells [16], but with dwindling neurogenic potential with brain development [75–78]. IGF2BP2 is expressed at a high level in neocortical NPCs at the early stage when these cells are proliferative and pluripotent, but to a less extent at the later stage when the cells lose their self-renewal ability [16]. It increases the neurogenic potential of NPCs and diverts them from astrocyte differentiation rather than regulating cell proliferation [16]. Thus, silencing IGF2BP2 expression inhibits neurogenesis while promoting NPC differentiation into glial cells [16]. A similar function of IGF2BP2 in stemness maintenance may also exist under pathological conditions; deletion of *IGF2BP2* gene in glioblastoma stem cells impairs their clonogenecity [30].

The driving mechanism behind the gradually diminishing expression of IGF2BP2 in favor of neurogenesis of NPCs in the early stage and glial cell differentiation in the later stage remains uncertain. Decreased expression of high-mobility group AT-hook 2 (HMGA2), which activates IGF2BP2 [79–82], induces similar but more prominent phenotypic changes in NPCs than IGF2BP2 overexpression, suggesting a potential HMGA2-IGF2BP2 axis during NPC self-renewal [16]. The downstream effector of IGF2BP2 in this setting is unknown; however, the known IGF2BP2 targets mediating focal adhesion remodeling and microtubule regulation may be involved in NPC2 cell stemness control and inhibition of differentiation [16].

#### 4.2.3. Metabolism

Among the mRNAs immunoprecipitated with IGF2BP2 ribonucleoprotein complexes in gliomaspheres formed by glioblastoma stem cells, genes regulating mitochondrial function and oxidative phosphorylation are significantly overrepresented [17]. Later isolation of mRNAs comigrating with polysomes obtained from the brown fat of mice with *IGF2BP2* deletion identified 13 transcripts encoding mitochondrial components [12]. These data link IGF2BP2 with the mitochondrial respiratory chain and cell metabolism. Although mitochondrial DNA provides easily accessible templates for the synthesis of its protein components, the majority of transcripts are translocated from the nucleus [83] by RBPs like IGF2BP2 which shuttles between the nucleus and mitochondria surface [17]. Inhibiting IGF2BP2 expression impairs the assembling and activities of mitochondrial respiratory complexes I and IV, suggesting essential roles of this IGF2BP2 in energy metabolism [17].

Significance of IGF2BP2 in metabolism has been confirmed by the phenotypic features demonstrated by mice with *IGF2BP2* deletion. The *IGF2BP2*-null mice gain less weight after birth and live longer than their wild-type littermates [12]. They are resistant to diet-induced obesity and fatty liver and exhibit better glucose tolerance and insulin sensitivity. Deletion of *IGF2BP2* leads to increased energy consumption and improved protection of the core temperature against coldness [12]. The brown fat cells from *IGF2BP2*-null mice exhibit a high level of UCP1, which functions as a cornerstone in thermogenesis by producing energy, maintaining body temperature, and reducing ATP synthesis [84]. Consistent with the negative regulation of UCP1 expression by IGF2BP2, the mTOR kinase complex, which activates IGF2BP2 and modulates the target mRNA translation [49], also inversely regulates the level of UCP1 [85–87], suggesting an mTOR-IGF2BP2-UCP1 cascade in energy metabolism. Apart from UCP1, IGF2 may be another effector of IGF2BP2 during metabolism. IGF2BP2 can be activated by mTOR and promotes its binding to IGF2 mRNA of IGF2 thereby leading to diabetes mellitus [12, 49]. These findings collectively highlight a significant role of IGF2BP2 in metabolism.

## 5. Regulation of IGF2BP2

**Figure 4:**
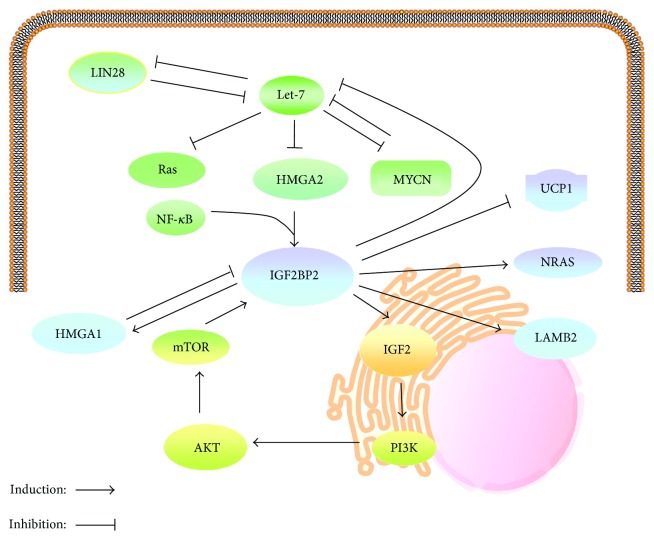
Regulation network of IGF2BP2. IGF2BP2 expression is controlled by HMGA2 which is a downstream target of Let-7 miRNA, and in return, IGF2BP2 binds to the Let-7 miRNA responsive elements and inhibits the Let-7 miRNA-mediated RNA degradation, independently of LIN28. HMGA1 suppresses expression of IGF2BP2, which in turn binds and stabilizes *HMGA1* mRNA. After phosphorylation mediated by mTOR, IGF2BP2 promotes translation of IGF2 by internal ribosomal entry and consequently the downstream PI3K/Akt signaling. IGF2BP2 may also regulate the expression of other downstream effectors such as UCP1, NRAS, and LAMB2 as well as the relevant cellular functions.

The functional significance of IGF2BPs in embryonic development, metabolism, and body growth determines that their levels must be tightly controlled in terms of timing and spatial distribution. High mobility group proteins are crucial for the expression of IGF2BP2. High mobility group protein A (HMGA) 1 suppresses expression of IGF2BP2, which in turn binds and stabilizes *HMGA1* mRNA. This feedback loop and other IGF2BP2 targets, such as IGF2, function collaboratively in promoting cell proliferation [88]. In *HMGA2*-null mice, IGF2BP2 expression was inhibited, supporting positive regulation of IGF2BP2 by HMGA2 (Figure 4) [89]. Later studies confirmed that HMGA2 promotes IGF2BP2 transcription by binding to the AT-rich region of the first intron of the *IGF2BP2* gene in cooperation with nuclear factor *κ*B (NF-*κ*B) [90]. Thus, it is unsurprising that the temporal expression profiles of HMGA2 and IGF2BP2 overlap substantially during mouse embryogenesis [89]. Interestingly, the *HMGA2* gene generates a truncated form of HMGA2 (HMGA2Tr), which is associated with tumorigenesis [91] and obesity [92, 93]. While HMGA2 increases *IGF2BP2* transcription, HMGA2Tr exhibits an inhibitory effect [89]. It is unknown whether the effects of the two HMGA2 isoforms on *IGF2BP2* transcription are associated with tumorigenesis.

The mediation of IGF2BP2 expression by HMGA2 has been proved to be critical for the expression of downstream targets of IGF2BP2, such as LAMB2, and Let-7 miRNA is involved in this cascade [10]. An increase of Let-7b inhibits HMGA2 expression which subsequently reduces IGF2BP2 and LAMB2 expression [10]. In a recent study, a photocatalytic activity-enhanced deoxyribose nucleotide cross-linking and immunoprecipitation (PAR-CLIP) assay demonstrated that IGF2BP2 bound to Let-7 miRNA recognition elements and suppressed the Let-7-mediated gene silencing independent of LIN28 proteins (Figure 4) [30], which are encoded by Let-7 target mRNAs, but inhibit the maturation of Let-7 [74]. Taken together, these results suggest that IGF2BP2 is regulated by a comprehensive network formed by HMGA2, Let-7/LIN28, IGF2, and other unidentified partners, in which IGF2BP2 itself is also involved in a feedback loop. Aberrant alterations in the expression of these genes are associated with cancer [94–96] and diabetic nephropathy [10].

Last but not least, IGF2BP2 activity can be mediated by mTOR, a major effector downstream of PI3K/Akt signaling [97]. In turn, a high level of IGF2BP2 enhances the expression of IGF2 which activates the PI3K/Akt/mTOR pathway and promotes the proliferation, migration, and epithelial-mesenchymal transition of cancer cells [98]. These results indicate a positive feedback loop between the PI3K/Akt/mTOR pathway and IGF2BP2 expression (Figure 4).

## 6. Association of IGF2BP2 with Cancer

Multitargeting RNA-binding is commonly seen for RBPs [99, 100], and the target transcripts of individual RNPs usually share a unique consensus motif as previously reported for IGF2BP1 [47] and other RBPs [99, 100]. Although the consensus binding site for IGF2BP2 has yet to be identified, hundreds of target mRNAs have been reported [17]. These mRNAs encode proteins engaged in a number of pathways of significant physiological importance [17]. Disruption of IGF2BP2 function, unsurprisingly, results in a variety of diseases and disorders such as diabetes and cancer (Table 2) [19].

### 6.1. Liver Cancer

The p62 isoform of IGF2BP2 was identified as an autoantigen in HCC as early as 1999 [35]. Autoantibody against p62 was found in 21% of HCC patients but completely absent in chronic hepatitis and liver cirrhosis, suggesting that the autoimmune response to IGF2BP2 is associated with transformation [35]. Later characterization showed that IGF2BP2 was expressed in scattered cells in liver cirrhosis nodules but present in all cells in HCC nodules [101], confirming the upregulation of IGF2BP2 with hepatocellular transformation. Consistent with these findings, immunohistochemistry analysis of the paraffin-embedded HCC suggests that about one-third of liver cancer patients have nodules expressing high levels of IGF2BP2 [102–105]. Of diagnostic and prognostic value, high IGF2BP2 expression in HCC predicts poor prognosis [27, 106]. Livers of mice overexpressing the IGF2BP2 splice variant p62 highly expressed the stem cell marker DLK1 and secreted DLK1 into the blood; DLK1was previously shown to correspond with poor survival in HCC [27].

How IGF2BP2 is mechanistically linked with hepatocellular transformation is unclear. The known target mRNA of this RBP, IGF2, has defined roles in cell division [107] and hepatocellular physiopathology [108] and is believed to drive cancer cell dedifferentiation and hepatocarcinogenesis [109]. Moreover, IGF2BP2 may also be involved in pathological conditions preceding HCC, such as nonalcoholic fatty liver disease (NAFLD) [110]. The presence of IGF2BP2 in liver cirrhosis [35, 105, 106] suggests that this RBP is generated during pathological alterations before liver transformation. More convincingly, mice with liver-specific overexpression of IGF2BP2 show more fat deposition and an earlier and more intense fibrogenesis than their wild-type littermates when they are fed a methionine-choline-deficient (MCD) diet [101], suggesting a driving role of IGF2BP2 in the pathogenesis of NAFLD [101]. Adipogenesis in the IGF2BP2 transgenic mice on MCD is stimulated by adipogenesis transcription factor Srebp1c [101], which also orchestrates adipogenesis and predicts a poor prognosis in HCC [111]. Furthermore, high IGF2BP2 expression in the liver of mice on MCD coincides with upregulation of connective tissue growth factor (CTGF) independent of transforming growth factor beta (TGF-*β*) [101]. The increase of CTGF could be induced by interleukin-13 (IL-13) [112] which is upregulated in IGF2BP2 transgenic mice [101]. Highlighting the importance of this IGF2BP2-IL13-CTGF axis in pathology, blockade of the IL-13 receptor in a mouse nonalcoholic steatohepatitis (NASH) model reduced fibrosis [113].

The pathological contribution of IGF2BP2 in NAFLD was speculated to be through monocyte chemoattractant protein-1 (MCP-1) [101] which is associated with NASH [101] and HCC [114]. In addition, IGF2BP2 may also modulate levels of tumor suppressor phosphatase and tensin homolog, affect activities of extracellular-signal regulated kinases, and contribute to the progression from NAFLD to HCC [101].

### 6.2. Glioblastoma

IGF2BP2 is overexpressed in glioblastoma compared to the normal adult brain [17, 30, 98], and its increase correlates with a poor prognosis in the proneural glioblastoma subtype, which is refractory to current therapy [17]. In *in vitro* 2-D and 3-D cultures of glioblastoma cancer stem cells (CSCs), IGF2BP2 is capable of binding to the mRNAs of 400 genes, which are overrepresented with genes regulating mitochondrial function and oxidative phosphorylation (OXPHOS) [17]. Some of these target transcripts encode proteins forming the mitochondrial respiratory chain complex. Furthermore, IGF2BP2 interacts with complex I (NADH: ubiquinone oxidoreductase) proteins. Silencing IGF2BP2 reduces the oxygen consumption rate of gliomaspheres and the activities of complexes I and IV and inhibits the clonogenicity *in vitro* and tumorigenicity in mice [17]. These data support the functions of IGF2BP2 in trafficking transcripts to mitochondria where it further mediates the assembly and activities of complexes I and IV in CSCs [17].

Previous *in vitro* study using glioblastoma cell lines suggests that IGF2BP2 promotes glioblastoma cell proliferation and invasion and facilitates their epithelial-mesenchymal transition [98]. Downregulating IGF2BP2 sensitizes cells to chemotherapy, likely through the inhibition of the PI3K/Akt signaling cascade [98], which is thought to be responsible for chemoresistance in many malignancies [115].

### 6.3. Colon Cancer

Therapeutic outcome of colon cancer and control of the relevant mortality relies on early diagnosis of disease [116]. Fecal occult bleeding, colonoscopic observation, and raised serum carcinoembryonic antigen (CEA) level remain commonly used and effective markers for the diagnosis of colon cancer [117–119]. However, these techniques are not ideal for large-scale colon cancer screening. Cancer-specific DNA, RNA, and proteins in body fluids are ideal candidate biomarkers for various cancers. Autoantibodies against cancer-specific autoantigens like IGF2BP2 represent a unique group of the new generation of biomarkers. In support of this, 23.4% of colon cancer patients were found with IGF2BP2 autoantibody in their blood whereas the percentages in patients with colon adenocarcinoma and healthy population were 4.8% and 2.9%, respectively [22]. The data indicate a gradual increase in the number of patients with an IGF2BP2 autoimmune response with cancer progression [22], indicating that IGF2BP2 may be associated with colon cancer transformation.

Immunohistochemistry demonstrates that 75% of patient tumors are positive for IGF2BP2, while normal colon tissues are completely negative [22]. It is unclear why only one-third of these patients produce an autoimmune response to this protein. Further studies in large cohorts of patients are required to dissect the correlation of IGF2BP2 autoimmune response with the stages and subcategories of colon cancer. Before this is achieved, autoantibody against IGF2BP2 may potentially be used together with other biomarkers, such as p53, CEA, c-myc, and antinuclear antibody, in the diagnosis of colon cancer [22, 120].

A recent *in vitro* study demonstrated experimentally that IGF2BP2 participates in the regulation of colon cancer cell proliferation. Exogenous IGF2BP2 promotes cell proliferation and survival whereas its suppression leads to inhibition of proliferation [29]. At the molecular level, IGF2BP2 protects RAF1 mRNA from the miR-195-mediated degradation [29]. RAF1 protein functions in the MAPK signaling cascade by phosphorylating and activating the MAPK kinases (MEK1/2), which further activates extracellular-related kinases (ERK1/2) and regulates cell survival and proliferation [29].

### 6.4. Breast Cancer

Immunohistochemistry demonstrates frequent elevation of IGF2BP2 in breast cancer patients. It is expressed in 66% of breast cancer tissues but is detectable in only 18% of normal breast tissues [24]. Importantly, a high level of its mRNA correlates with a short survival [26], suggesting a potential prognostic value of IGF2BP2 in breast cancer. IGF2BP2 can be particularly informative for basal-like breast cancer, a subtype mostly associated with triple-negative breast cancer, which generally has a poor survival [121].

In concord with IGF2BP2 elevation in tumor tissue, autoantibody against IGF2BP2 is detected in 14.3% of blood samples from breast cancer patients, compared to only 5.6% in patients with benign tumors and 2.2% in healthy individuals [24]. The occurrence of this autoimmune response, easily assessable by liquid biopsy, can be used in stratification of breast cancer and, potentially, in the prognosis of certain subtypes of breast cancer.

### 6.5. Gastrointestinal Cancer

In a study of 144 cases of gastrointestinal cancer including 32 esophageal, 66 gastric, and 46 large intestine cancers, Su et al. demonstrated that all cancer tissues expressed IGF2BP2 [122]. Among 83 serum samples from 48 gastric, 10 esophageal, and 25 large intestine cancer patients analyzed, 32 were detected with IGF2BP2 autoantibody [122]. The frequency of autoimmune response to IGF2BP2 was significantly higher in patients with metastatic tumors than in those with local disease [122]. Consistently, high IGF2BP2 expression in esophageal adenocarcinoma was found in another independent study, particularly in tumors of increased size and in metastatic lesions, suggesting the potential prognostic value of this protein [25].

Alterations in the *IGF2BP2* gene may have potential prognostic value in gastrointestinal cancer. In gastric cancer patients treated by first-line combinational therapy with epirubicin, oxaliplatin, and 5-fluorouracil, *IGF2BP2* polymorphisms rs4402960 and rs6769511 are less common in patients with disease progression than in those with controlled disease [28]. Whether these nucleotide changes improve the response of gastric cancer patients to chemotherapies remains to be confirmed in future studies.

### 6.6. Endometrial Carcinoma

Histological classification of endometrioid and serous subtypes of endometrial carcinoma, which are different in terms of pathogenesis, clinical characteristics, prognosis, and treatments, can be challenging [123]. Immunohistochemical analysis for the recommended markers, such as p53, Ki67, and p16 [124, 125], can be of limited help in some difficult cases [123], suggesting that more definitive markers are needed. In a study involving 320 endometrial biopsy cases, immunohistochemical analysis indicated that IGF2BP2 was expressed in all proliferative and inactive endometrial glandular cells in both normal and serous carcinoma tissues. However, IGF2BP2 expression was lost in all endometrioid cases, by 25% to >95% of tumor cell populations [123]. More IGF2BP2 loss was observed in low-grade tumors [123]. These data suggest the potential of this protein as a biomarker for endometrioid endometrial cancer.

### 6.7. Sarcomas

Several lines of evidence support potential roles of IGF2BP2 in sarcomas. A higher level of IGF2BP2 was found in well-differentiated liposarcoma, in comparison with myxoid liposarcoma [90], suggesting that IGF2BP2 may potentially be used to distinguish myxoid liposarcoma from the well-differentiated liposarcoma. The expression of IGF2BP2 in this setting is driven by HMGA2 and NF-*κ*B, through binding to their regulatory elements tandemly located in the first intron of the *IGF2BP2* gene [90]. In embryonic rhabdomyosarcoma, NRAS is frequently mutated and essential for tumor maintenance. The HMGA2-driven IGF2BP2 expression is fundamental for the stability of NRAS mRNA and proteins, suggesting the importance of the HMGA-IGF2BP2 axis in different sarcomas.

### 6.8. Other Carcinomas

A few studies have also shown the association of IGF2BP2 with other malignancies, including prostate, ovarian, and testicular cancer. An enzyme-linked immunosorbent assay (ELISA) screening of 34 serum samples from ovarian cancer patients using 89 serum specimens from healthy donors as controls detected IGF2BP2 autoantibody in 29.4% of ovarian cancer patient, significantly higher than the detection rate in the healthy donors (1.1%) [23]. Interestingly, the autoantibody against IGF2BP1 was also detected at a comparable percentage (26.5%), and it is unknown whether the autoimmune responses to IGF2BP1 and IGF2BP2 are correlated in ovarian cancer patients.

In a small cohort of patients with testicular tumors originating from germ cells (30 cases) and somatic cells (3 cases), immunoreactivities of IGF2BP1, IGF2BP2, and IGF2BP3 were frequently observed but with different patterns. While IGF2BP1 was detected in all carcinomas, IGF2BP2 and IGF2BP3 were only seen in a subset of testicular cancers. These findings are, however, inconclusive due to the limited patient numbers [72], and future studies are needed to characterize the specificities and potential diagnostic values of IGF2BP2 and IGF2BP3 expression for different subtypes of testicular cancer.

## 7. IGF2BP2 and Cancer Stem Cells

Cancer stem cells are a small group of cells that are capable of reinitiating tumors and believed to be the driving force behind the tumor growth, cancer recurrence, metastasis, and chemoresistance [126, 127]. Targeting these cells, especially their plasticity and self-renewal, represents one of the major efforts in the past two decades to eradicate tumor cells. IGF2BPs have frequently been shown to be associated with CSCs in different malignancies. IGF2BP1 promotes the enrichment and survival of a population of CD24^+^CD44^+^ tumor-initiating cells in colorectal cancer [128]. IGF2BP3 is upregulated in tumor-initiating CD133^+^CD49f^+^ cells in mouse HCC and promotes their pluripotency and tumorigenesis by inhibiting TGF-*β* tumor suppressor pathway [129]. It is also specifically overexpressed in mixed lineage leukemia-rearranged (MLL-rearranged) B-acute lymphoblastic leukemia (B-ALL) in which it mediates the proliferation of hematopoietic stem and progenitor cells [130].

Multiple lines of evidence from independent studies have linked IGF2BP2 with CSCs. As aforementioned, IGF2BP2 mediates the growth of gliomaspheres formed by glioblastoma stem cells by controlling OXPHOS through dozens of genes involved in mitochondria activity [17]. The short isoform of IGF2BP2, p62, is overexpressed in HCC with stem-like features and hypervascularization, and the livers of p62 transgenic mice highly express the stem cell marker DLK1 and generate tumors with more aggressive and stem-like phenotype [27]. Consistent with these observations in CSCs are the determinant roles of IGF2BP2 in the differentiation of NPCs [16] and myoblasts [131, 132].

Mechanistically, IGF2BP2 induces genomic instability and enhances reactive oxygen species production in HCC CSCs [27]. In glioblastoma CSCs, IGF2BP2 regulates cell metabolism. Moreover, IGF2BP2 binds to the Let-7 miRNA recognition elements and protects the target mRNAs from the Let-7-mediated silencing, independent of LIN28 [30]. This interrupts the differentiation induced by tumor suppressor Let-7 miRNA and maintains the self-renewal and tumor-initiating capacities of CSCs [30]. IGF2BP2 also functions downstream of long noncoding RNA HIF1A-AS2 (hypoxia-inducible factor 1 *α*-antisense RNA 2), via maintaining HMGA1 expression, and mediates the growth of tumors formed by glioblastoma CSCs [133]. These findings are believed to be a tip of the iceberg of IGF2BP2 function in CSCs; the full image is yet to be accomplished and pathways involved remain to be understood.

## 8. IGF2BP2, Diabetes, and Cancer

Patients with diabetes (predominantly type 2) generally have a higher incidence of many cancers, such as liver, pancreas, endometrium, colon, breast, and bladder cancer, as well as increased cancer-related mortality compared to the nondiabetic population [134–137]. Hyperglycemia, hyperinsulinemia, and dysregulation of IGFs and adipokines are among the major mechanisms that facilitate tumorigenesis and cancer progression [137]. The genetic and molecular drivers behind these functional aberrations represent a group of ideal candidates which can be targeted to block the bioenergetic sources of cancer cells.

The insulin/IGF axis tops the list of potential mechanisms that link diabetes and cancer as reviewed elsewhere [134, 135]. The contribution of IGF2BP2 to this lethal link is largely unknown. A major member of the IGF family of growth factors, IGF2, is regulated by IGF2BP2 posttranscriptionally [49] and commonly dysregulated in type 2 diabetes [138] along with IGF2BP2 itself [19]. Elevated IGF2 predisposes patients to diabetes [138] and is believed to be involved in the association between diabetes and increased breast cancer incidence in African-American women [139]. In this setting, the level of IGF2BP2 can be mediated by the concentration of glucose, which controls the upstream regulators of IGF2BP2, Let-7, and HMGA2 [10]. After phosphorylation by mTOR, the activated IGF2BP2 further enhances the expression of IGF2 [49].

Notably, type 2 diabetes and multiple cancers share common genetic fingerprints in the *IGF2BP2* gene. The *IGF2BP2* rs4402960 polymorphism, which conveys an increased risk of type 2 diabetes [20, 140–143], also increases the risks of colon and breast cancer according to the results from independent studies [21, 144]. However, an inverse correlation between this polymorphism and prostate cancer was reported [145]. In gastric cancer, *IGF2BP2* rs4402960 and rs6769511 are more often detected in patients responsive to a combinational chemotherapy of epirubicin, oxaliplatin, and 5-fluorouracil [28]. These findings highlight the importance of these polymorphisms of *IGF2BP2* in diabetes and cancer development; however, how the genetic variations affect the expression of IGF2BP2 and IGF2 is uncertain, and their functional consequences in diabetes and cancer progression require further characterization.

## 9. Conclusions and Perspectives

IGF2BP2 belongs to a large family of RBPs that coordinate the export, trafficking, and precise localization and translation of RNA in cells [4]. The tandem repeats of two RRMs and four KHs spaced by unique linker regions in its structure allow it to bind to a subset of transcripts and proteins and play multifaceted roles in cellular maintenance and embryonic development. In support of this, adult *IGF2BP2*-null mice are lean and small in size but are highly resistant to diet-induced obesity and are long lived compared to their normal littermates [12], suggesting critical roles of IGF2BP2 in metabolism although the detailed mechanism remains to be investigated.

During embryonic development and in adults, 3 IGF2BPs exhibit different expression patterns suggesting their different physiological duties. Indeed, loss-of-function mouse models for IGF2BP1 and IGF2BP2 demonstrate dramatically different phenotypic features. Meanwhile, these proteins share a high level of identity in amino acid sequences and structures, suggesting a possible functional redundancy between them. In screening for the targets of IGF2BPs in human pluripotent stem cells using eCLIP, IGF2BP2 was found to share more targets with IGF2BP1, in comparison with IGF2BP3, even though the latter was believed to be more relevant to IGF2BP1 [146]. These data further suggest the functional redundancies between IGF2BPs although the extents and their biological significance remain to be characterized in future studies.

A number of studies have demonstrated elevated expression or de novo synthesis of IGF2BP2 in a range of malignancies. These alterations, and the staining patterns of IGF2BP2 in some cancer types, can be unique to cancer cells or a subcategory of cancer and potentially used as a diagnostic biomarker, for example, in differentiating the endometrioid subtype of endometrial cancer from normal endometrium and serous endometrial carcinoma [123]. Moreover, the incidence of autoimmune response to IGF2BP2 is substantially more frequent in cancerous than in normal tissue [22, 24, 35, 122], implicating that detection of autoantibody against IGF2BP2 in serum may assist patient diagnosis and stratification. Noticeably, the cohorts of patients used in these studies were mostly small; future multi-institutional and multinational large-scale characterizations are required to define the extent and specificity of IGF2BP2 expression and IGF2BP2 autoimmunity in different types of cancer.

The experimental evidence accumulated so far collectively supports a procancerous role of IGF2BP2 in cancer progression. Firstly, IGF2BP2 functions as a tumor promoter [88]. Its amplification and overexpression in many cancers correlate with cancer progression and predict poor prognosis. Secondly, IGF2BP2 promotes cancer cell survival, proliferation, and migration although the underlying mechanisms remain poorly defined [28, 29, 39]. Thirdly, *in vitro* and *in vivo* studies have provided solid evidence that IGF2BP2 is a dominant factor in the self-renewal of CSCs [17]. Fourthly, IGF2BP2 regulates the processing of transcripts that encode key subunits in mitochondrial respiratory chain complexes, as well as the assembly of these complexes, and fine-tunes oxidative phosphorylation in CSCs [17]. In addition, IGF2BP2 exhibits counteracting roles against tumor suppressor Let-7 miRNA by preventing the Let-7-mediated RNA degradation [10, 30]. Future studies are required to dissect the mechanisms behind the multifaceted rules of IGF2BP2 in cancer and evaluate the potential of targeting this protein in therapeutic development. Of particular interest is whether IGF2BP2 can be suppressed to divert CSCs towards differentiation or sensitize them to chemotherapy so as to reduce the recurrence and metastasis of cancer.

Associations of metabolic diseases such as diabetes and obesity with the incidence, progression, and clinical outcome have been commonly recognized [134–137]. Delineation of the mechanisms that position cancer cells advantageously in terms of nutrition and energy supply is a prerequisite for us to develop approaches to block the signaling and energy supply to cancer cells. IGF2BP2 is likely to participate in these mechanisms. In one aspect, IGF2BP2 functions in insulin-induced signaling and metabolism, and depletion of IGF2BP2 in mice leads to glucose tolerance, insulin sensitivity, increased energy expenditure, slower body growth, and resistance to diet-induced obesity and fatty liver [12]. In another aspect, IGF2BP2 upregulation correlates with malignant transformation in liver and is required for the maintenance of stemness of CSCs. It will be important to characterize whether mice with *IGF2BP2* deletion are more resistant to spontaneous tumorigenesis.

## Figures and Tables

**Table 1:** Known target mRNAs of IGF2BP2.

Target	Effect on translation	Physiological function	Reference
IGF2	Enhancement	Growth, metabolism	[9, 49]
UCP	Inhibition	Metabolism	[12]
PINCH2	Enhancement	Glomerular stability	[10]
PINCH2	Inhibition	Cell migration and adhesion	[11]
MURF3	Enhancement	Myoblast microtubule stability	[11]
CI/CIV	Enhancement	Myoblast microtubule stability	[17]
NRAS	Enhancement	Rhabdomyosarcoma	[147]
HMGA1	Enhancement	Cell proliferation	[88]

**Table 2:** Expression of IGF2BP2 and IGF2BP2-autoantibody (Ab) in human cancers.

Cancer	Incidence		Reference
	IGF2BP2 (%)	IGF2BP2-Ab (%)	
Glioblastoma	78.4%	—	[17]
Hepatocellular carcinoma	33.3%	21.0%	[35]
Gastric cancer	44.0%	76.5%	[122]
Colon cancer	44.0%	21.7%	[120]
Breast cancer	66%	14.3%	[148]
Endometrial carcinoma	25%	—	[123]
Oophoroma	—	29.4%	[72]
Testicular cancer	87.6%	—	[23]
Liposarcoma	78.6%	—	[90]

## References

[1] Wozniak M., Dus-Szachniewicz K., Ziolkowski P. (2015). Insulin-like growth factor-2 is induced following 5-aminolevulinic acid-mediated photodynamic therapy in SW620 human colon cancer cell line. *International Journal of Molecular Sciences*.

[2] Bergman D., Halje M., Nordin M., Engstrom W. (2013). Insulin-like growth factor 2 in development and disease: a mini-review. *Gerontology*.

[3] Sasaki H., Ishihara K., Kato R. (2000). Mechanisms of *Igf2/H19* imprinting: DNA methylation, chromatin and long-distance gene regulation. *The Journal of Biochemistry*.

[4] Lunde B. M., Moore C., Varani G. (2007). RNA-binding proteins: modular design for efficient function. *Nature Reviews Molecular Cell Biology*.

[5] Glisovic T., Bachorik J. L., Yong J., Dreyfuss G. (2008). RNA-binding proteins and post-transcriptional gene regulation. *FEBS Letters*.

[6] Nielsen F. C., Nielsen J., Christiansen J. (2001). A family of IGF-II mRNA binding proteins (IMP) involved in RNA trafficking. *Scandinavian Journal of Clinical and Laboratory Investigation*.

[7] Yaniv K., Yisraeli J. K. (2002). The involvement of a conserved family of RNA binding proteins in embryonic development and carcinogenesis. *Gene*.

[8] Yisraeli J. K. (2005). VICKZ proteins: a multi-talented family of regulatory RNA-binding proteins. *Biology of the Cell*.

[9] Nielsen J., Christiansen J., Lykke-Andersen J., Johnsen A. H., Wewer U. M., Nielsen F. C. (1999). A family of insulin-like growth factor II mRNA-binding proteins represses translation in late development. *Molecular Cellular Biology*.

[10] Schaeffer V., Hansen K. M., Morris D. R., LeBoeuf R. C., Abrass C. K. (2012). RNA-binding protein IGF2BP2/IMP2 is required for laminin-*β*2 mRNA translation and is modulated by glucose concentration. *American Journal of Physiology Renal Physiology*.

[11] Boudoukha S., Cuvellier S., Polesskaya A. (2010). Role of the RNA-binding protein IMP-2 in muscle cell motility. *Molecular and Cellular Biology*.

[12] Dai N., Zhao L., Wrighting D. (2015). IGF2BP2/IMP2-deficient mice resist obesity through enhanced translation of *Ucp1* mRNA and other mRNAs encoding mitochondrial proteins. *Cell Metabolism*.

[13] Rodriguez A., Durán A., Selloum M. (2006). Mature-onset obesity and insulin resistance in mice deficient in the signaling adapter p62. *Cell Metabolism*.

[14] Ruchat S. M., Elks C. E., Loos R. J. F. (2009). Association between insulin secretion, insulin sensitivity and type 2 diabetes susceptibility variants identified in genome-wide association studies. *Acta Diabetologica*.

[15] Zhang J., Chan E. K. (2002). Autoantibodies to IGF-II mRNA binding protein p62 and overexpression of p62 in human hepatocellular carcinoma. *Autoimmunity Reviews*.

[16] Fujii Y., Kishi Y., Gotoh Y. (2013). IMP2 regulates differentiation potentials of mouse neocortical neural precursor cells. *Genes to Cells*.

[17] Janiszewska M., Suva M. L., Riggi N. (2012). Imp2 controls oxidative phosphorylation and is crucial for preserving glioblastoma cancer stem cells. *Genes & Development*.

[18] Wu Y., Li H., Loos R. J. F. (2008). Common variants in *CDKAL1*, *CDKN2A/B*, *IGF2BP2*, *SLC30A8*, and *HHEX/IDE* genes are associated with type 2 diabetes and impaired fasting glucose in a Chinese Han population. *Diabetes*.

[19] Christiansen J., Kolte A. M., Hansen T.v O., Nielsen F. C. (2009). IGF2 mRNA-binding protein 2: biological function and putative role in type 2 diabetes. *Journal of Molecular Endocrinology*.

[20] Huang Q., Yin J., Dai X. (2010). *IGF2BP2* variations influence repaglinide response and risk of type 2 diabetes in Chinese population. *Acta Pharmacologica Sinica*.

[21] Liu G., Zhu T., Cui Y. (2015). Correlation between *IGF2BP2* gene polymorphism and the risk of breast cancer in Chinese Han women. *Biomedicine & Pharmacotherapy*.

[22] Liu W., Li Z., Xu W., Wang Q., Yang S. (2013). Humoral autoimmune response to IGF2 mRNA-binding protein (IMP2/p62) and its tissue-specific expression in colon cancer. *Scandinavian Journal of Immunology*.

[23] Liu X., Ye H., Li L., Li W., Zhang Y., Zhang J. Y. (2014). Humoral autoimmune responses to insulin-like growth factor II mRNA-binding proteins IMP1 and p62/IMP2 in ovarian cancer. *Journal of Immunology Research*.

[24] Liu W., Li Y., Wang B., Dai L., Qian W., Zhang J. Y. (2015). Autoimmune response to IGF2 mRNA-binding protein 2 (IMP2/p62) in breast cancer. *Scandinavian Journal of Immunology*.

[25] Barghash A., Golob-Schwarzl N., Helms V., Haybaeck J., Kessler S. M. (2016). Elevated expression of the *IGF2* mRNA binding protein 2 (IGF2BP2/IMP2) is linked to short survival and metastasis in esophageal adenocarcinoma. *Oncotarget*.

[26] Barghash A., Helms V., Kessler S. M. (2015). Overexpression of *IGF2* mRNA-binding protein 2 (IMP2/p62) as a feature of basal-like breast cancer correlates with short survival. *Scandinavian Journal of Immunology*.

[27] Kessler S. M., Laggai S., Barghash A. (2015). IMP2/p62 induces genomic instability and an aggressive hepatocellular carcinoma phenotype. *Cell Death & Disease*.

[28] Liu X., Chen Z., Zhao X. (2015). Effects of *IGF2BP2*, *KCNQ1* and *GCKR* polymorphisms on clinical outcome in metastatic gastric cancer treated with EOF regimen. *Pharmacogenomics*.

[29] Ye S., Song W., Xu X., Zhao X., Yang L. (2016). IGF2BP2 promotes colorectal cancer cell proliferation and survival through interfering with *RAF-1* degradation by miR-195. *FEBS Letters*.

[30] Degrauwe N., Schlumpf T. B., Janiszewska M. (2016). The RNA binding protein IMP2 preserves glioblastoma stem cells by preventing let-7 target gene silencing. *Cell Reports*.

[31] Gaynes J. A., Otsuna H., Campbell D. S., Manfredi J. P., Levine E. M., Chien C. B. (2015). The RNA binding protein Igf2bp1 is required for zebrafish RGC axon outgrowth *in vivo*. *PLoS One*.

[32] Doyle G. A. R., Leeds P. F., Fleisig A. J., Ross J., Betz N. A., Prokipcak R. D. (1998). The *c-myc* coding region determinant-binding protein: a member of a family of KH domain RNA-binding proteins. *Nucleic Acids Research*.

[33] Havin L., Git A., Elisha Z. (1998). RNA-binding protein conserved in both microtubule- and microfilament-based RNA localization. *Genes & Development*.

[34] Mowry K., Melton D. (1992). Vegetal messenger RNA localization directed by a 340-nt RNA sequence element in Xenopus oocytes. *Science*.

[35] Zhang J.-Y., Chan E. K. L., Peng X.-X., Tan E. M. (1999). A novel cytoplasmic protein with RNA-binding motifs is an autoantigen in human hepatocellular carcinoma. *Journal of Experimental Medicine*.

[36] Raedle J., Oremek G., Truschnowitsch M. (1998). Clinical evaluation of autoantibodies to p53 protein in patients with chronic liver disease and hepatocellular carcinoma. *European Journal of Cancer*.

[37] He Y., Brown M. A., Rothnagel J. A., Saunders N. A., Smith R. (2005). Roles of heterogeneous nuclear ribonucleoproteins A and B in cell proliferation. *Journal of Cell Science*.

[38] He Y., Smith R. (2009). Nuclear functions of heterogeneous nuclear ribonucleoproteins A/B. *Cellular and Molecular Life Sciences*.

[39] Bell J. L., Wächter K., Mühleck B. (2013). Insulin-like growth factor 2 mRNA-binding proteins (IGF2BPs): post-transcriptional drivers of cancer progression?. *Cellular and Molecular Life Sciences*.

[40] Git A., Standart N. (2002). The KH domains of *Xenopus* Vg1RBP mediate RNA binding and self-association. *RNA*.

[41] Amarasinghe A. K., MacDiarmid R., Adams M. D., Rio D. C. (2001). An in vitro-selected RNA-binding site for the KH domain protein PSI acts as a splicing inhibitor element. *RNA*.

[42] Griffin D., Penberthy W. T., Lum H., Stein R. W., Taylor W. L. (2003). Isolation of the B3 transcription factor of the *Xenopus* TFIIIA gene. *Gene*.

[43] Farina K. L., Huttelmaier S., Musunuru K., Darnell R., Singer R. H. (2003). Two ZBP1 KH domains facilitate *β*-actin mRNA localization, granule formation, and cytoskeletal attachment. *Journal of Cell Biology*.

[44] Nielsen F. C., Nielsen J., Kristensen M. A., Koch G., Christiansen J. (2002). Cytoplasmic trafficking of IGF-II mRNA-binding protein by conserved KH domains. *Journal of Cell Science*.

[45] Jensen K. B., Musunuru K., Lewis H. A., Burley S. K., Darnell R. B. (2000). The tetranucleotide UCAY directs the specific recognition of RNA by the Nova K-homology 3 domain. *Proceedings of the National Academy of Sciences of the United States of America*.

[46] Atlas R., Behar L., Elliott E., Ginzburg I. (2004). The insulin-like growth factor mRNA binding-protein IMP-1 and the Ras-regulatory protein G3BP associate with tau mRNA and HuD protein in differentiated P19 neuronal cells. *Journal of Neurochemistry*.

[47] Patel G. P., Bag J. (2006). IMP1 interacts with poly(A)-binding protein (PABP) and the autoregulatory translational control element of PABP-mRNA through the KH III-IV domain. *The FEBS Journal*.

[48] Nielsen J., Kristensen M. A., Willemoes M., Nielsen F. C., Christiansen J. (2004). Sequential dimerization of human zipcode-binding protein IMP1 on RNA: a cooperative mechanism providing RNP stability. *Nucleic Acids Research*.

[49] Dai N., Rapley J., Angel M., Yanik M. F., Blower M. D., Avruch J. (2011). mTOR phosphorylates IMP2 to promote IGF2 mRNA translation by internal ribosomal entry. *Genes & Development*.

[50] Dai N., Christiansen J., Nielsen F. C., Avruch J. (2013). mTOR complex 2 phosphorylates IMP1 cotranslationally to promote IGF2 production and the proliferation of mouse embryonic fibroblasts. *Genes & Development*.

[51] Hüttelmaier S., Zenklusen D., Lederer M. (2005). Spatial regulation of *β*-actin translation by Src-dependent phosphorylation of ZBP1. *Nature*.

[52] Leung K. M., van Horck F. P. G., Lin A. C., Allison R., Standart N., Holt C. E. (2006). Asymmetrical *β*-actin mRNA translation in growth cones mediates attractive turning to netrin-1. *Nature Neuroscience*.

[53] Yao J., Sasaki Y., Wen Z., Bassell G. J., Zheng J. Q. (2006). An essential role for *β*-actin mRNA localization and translation in Ca^2+^-dependent growth cone guidance. *Nature Neuroscience*.

[54] Nielsen J., Adolph S. K., Rajpert-De Meyts E. (2003). Nuclear transit of human zipcode-binding protein IMP1. *Biochemical Journal*.

[55] Oleynikov Y., Singer R. H. (2003). Real-time visualization of ZBP1 association with *β*-actin mRNA during transcription and localization. *Current Biology*.

[56] Johnson C. D., Esquela-Kerscher A., Stefani G. (2007). The *let-7* microRNA represses cell proliferation pathways in human cells. *Cancer Research*.

[57] Weidensdorfer D., Stohr N., Baude A. (2009). Control of c-myc mRNA stability by IGF2BP1-associated cytoplasmic RNPs. *RNA*.

[58] Pan F., Huttelmaier S., Singer R. H., Gu W. (2007). ZBP2 facilitates binding of ZBP1 to *β*-actin mRNA during transcription. *Molecular and Cellular Biology*.

[59] Deshler J. O., Highett M. I., Abramson T., Schnapp B. J. (1998). A highly conserved RNA-binding protein for cytoplasmic mRNA localization in vertebrates. *Current Biology*.

[60] Stöhr N., Lederer M., Reinke C. (2006). ZBP1 regulates mRNA stability during cellular stress. *Journal of Cell Biology*.

[61] Zhang H. L., Eom T., Oleynikov Y. (2001). Neurotrophin-induced transport of a *β*-actin mRNP complex increases *β*-actin levels and stimulates growth cone motility. *Neuron*.

[62] Git A., Allison R., Perdiguero E., Nebreda A. R., Houliston E., Standart N. (2009). Vg1RBP phosphorylation by Erk2 MAP kinase correlates with the cortical release of Vg1 mRNA during meiotic maturation of *Xenopus* oocytes. *RNA*.

[63] Moraes K. C., Quaresma A. J., Maehnss K., Kobarg J. (2003). Identification and characterization of proteins that selectively interact with isoforms of the mRNA binding protein AUF1 (hnRNP D). *Biological Chemistry*.

[64] Runge S., Nielsen F. C., Nielsen J., Lykke-Andersen J., Wewer U. M., Christiansen J. (2000). H19 RNA binds four molecules of insulin-like growth factor II mRNA-binding protein. *The Journal of Biological Chemistry*.

[65] Adolph S. K., DeLotto R., Nielsen F. C., Christiansen J. (2009). Embryonic expression of *Drosophila* IMP in the developing CNS and PNS. *Gene Expression Patterns*.

[66] Mueller-Pillasch F., Pohl B., Wilda M. (1999). Expression of the highly conserved RNA binding protein KOC in embryogenesis. *Mechanisms of Development*.

[67] Nielsen J., Cilius Nielsen F., Kragh Jakobsen R., Christiansen J. (2000). The biphasic expression of IMP/Vg1-RBP is conserved between vertebrates and *Drosophila*. *Mechanisms of Development*.

[68] Hansen T. V. O., Hammer N. A., Nielsen J. (2004). Dwarfism and impaired gut development in insulin-like growth factor II mRNA-binding protein 1-deficient mice. *Molecular and Cellular Biology*.

[69] Mori H., Sakakibara S., Imai T. (2001). Expression of mouse *igf2* mRNA-binding protein 3 and its implications for the developing central nervous system. *Journal of Neuroscience Research*.

[70] Wang T., Fan L., Watanabe Y. (2003). L523S, an RNA-binding protein as a potential therapeutic target for lung cancer. *British Journal of Cancer*.

[71] Gu L., Shigemasa K., Ohama K. (2004). Increased expression of IGF II mRNA-binding protein 1 mRNA is associated with an advanced clinical stage and poor prognosis in patients with ovarian cancer. *International Journal of Oncology*.

[72] Hammer N. A., Hansen T.v O., Byskov A. G. (2005). Expression of IGF-II mRNA-binding proteins (IMPs) in gonads and testicular cancer. *Reproduction*.

[73] Boylan K. L. M., Mische S., Li M. (2008). Motility screen identifies *Drosophila* IGF-II mRNA-binding protein—zipcode-binding protein acting in oogenesis and synaptogenesis. *PLoS Genetics*.

[74] Nishino J., Kim S., Zhu Y., Zhu H., Morrison S. J. (2013). A network of heterochronic genes including *Imp1* regulates temporal changes in stem cell properties. *eLife*.

[75] Hirabayashi Y., Gotoh Y. (2005). Stage-dependent fate determination of neural precursor cells in mouse forebrain. *Neuroscience Research*.

[76] Hirabayashi Y., Gotoh Y. (2010). Epigenetic control of neural precursor cell fate during development. *Nature Reviews Neuroscience*.

[77] Qian X., Shen Q., Goderie S. K. (2000). Timing of CNS cell generation: a programmed sequence of neuron and glial cell production from isolated murine cortical stem cells. *Neuron*.

[78] Temple S. (2001). The development of neural stem cells. *Nature*.

[79] Kishi Y., Fujii Y., Hirabayashi Y., Gotoh Y. (2012). HMGA regulates the global chromatin state and neurogenic potential in neocortical precursor cells. *Nature Neuroscience*.

[80] Naka H., Nakamura S., Shimazaki T., Okano H. (2008). Requirement for COUP-TFI and II in the temporal specification of neural stem cells in CNS development. *Nature Neuroscience*.

[81] Nishino J., Kim I., Chada K., Morrison S. J. (2008). Hmga2 promotes neural stem cell self-renewal in young but not old mice by reducing p16^Ink4a^ and p19^Arf^ expression. *Cell*.

[82] Sanosaka T., Namihira M., Asano H. (2008). Identification of genes that restrict astrocyte differentiation of midgestational neural precursor cells. *Neuroscience*.

[83] Suissa M., Schatz G. (1982). Import of proteins into mitochondria. Translatable mRNAs for imported mitochondrial proteins are present in free as well as mitochondria-bound cytoplasmic polysomes. *The Journal of Biological Chemistry*.

[84] Bonet M. L., Mercader J., Palou A. (2017). A nutritional perspective on UCP1-dependent thermogenesis. *Biochimie*.

[85] Zoncu R., Efeyan A., Sabatini D. M. (2011). mTOR: from growth signal integration to cancer, diabetes and ageing. *Nature Reviews Molecular Cell Biology*.

[86] Liao C. Y., Anderson S. S., Chicoine N. H. (2016). Rapamycin reverses metabolic deficits in Lamin A/C-deficient mice. *Cell Reports*.

[87] Wang Y., Li Z., Zhang X. (2016). Nesfatin-1 promotes brown adipocyte phenotype. *Scientific Reports*.

[88] Dai N., Ji F., Wright J., Minichiello L., Sadreyev R., Avruch J. (2017). IGF2 mRNA binding protein-2 is a tumor promoter that drives cancer proliferation through its client mRNAs IGF2 and HMGA1. *eLife*.

[89] Brants J. R., Ayoubi T. A. Y., Chada K., Marchal K., Van de Ven W. J. M., Petit M. M. R. (2004). Differential regulation of the insulin-like growth factor II mRNA-binding protein genes by architectural transcription factor HMGA2. *FEBS Letters*.

[90] Cleynen I., Brants J. R., Peeters K. (2007). HMGA2 regulates transcription of the *Imp2* gene via an intronic regulatory element in cooperation with nuclear factor-*κ*B. *Molecular Cancer Research*.

[91] Fedele M., Berlingieri M. T., Scala S. (1998). Truncated and chimeric *HMGI-C* genes induce neoplastic transformation of NIH3T3 murine fibroblasts. *Oncogene*.

[92] Arlotta P., Tai A. K.-F., Manfioletti G., Clifford C., Jay G., Ono S. J. (2000). Transgenic mice expressing a truncated form of the high mobility group I-C protein develop adiposity and an abnormally high prevalence of lipomas. *The Journal of Biological Chemistry*.

[93] Battista S., Fidanza V., Fedele M. (1999). The expression of a truncated *HMGI-C* gene induces gigantism associated with lipomatosis. *Cancer Research*.

[94] Lee Y. S., Dutta A. (2007). The tumor suppressor microRNA *let-7* represses the HMGA2 oncogene. *Genes & Development*.

[95] Motoyama K., Inoue H., Nakamura Y., Uetake H., Sugihara K., Mori M. (2008). Clinical significance of high mobility group A2 in human gastric cancer and its relationship to *let-7* microRNA family. *Clinical Cancer Research*.

[96] Mu G., Liu H., Zhou F. (2010). Correlation of overexpression of HMGA1 and HMGA2 with poor tumor differentiation, invasion, and proliferation associated with let-7 down-regulation in retinoblastomas. *Human Pathology*.

[97] Lai B. Q., Che M. T., Du B. L. (2016). Transplantation of tissue engineering neural network and formation of neuronal relay into the transected rat spinal cord. *Biomaterials*.

[98] Mu Q., Wang L., Yu F. (2015). Imp2 regulates GBM progression by activating IGF2/PI3K/Akt pathway. *Cancer Biology & Therapy*.

[99] Lerga A., Hallier M., Delva L. (2001). Identification of an RNA binding specificity for the potential splicing factor TLS. *The Journal of Biological Chemistry*.

[100] Edwards J., Malaurie E., Kondrashov A. (2011). Sequence determinants for the tandem recognition of UGU and CUG rich RNA elements by the two N—terminal RRMs of CELF1. *Nucleic Acids Research*.

[101] Simon Y., Kessler S. M., Bohle R. M., Haybaeck J., Kiemer A. K. (2014). The insulin-like growth factor 2 (*IGF2*) mRNA-binding protein p62/IGF2BP2-2 as a promoter of NAFLD and HCC?. *Gut*.

[102] Covini G., von Muhlen C. A., Pacchetti S., Colombo M., Chan E. K. L., Tan E. M. (1997). Diversity of antinuclear antibody responses in hepatocellular carcinoma. *Journal of Hepatology*.

[103] Imai H., Nakano Y., Kiyosawa K., Tan E. M. (1993). Increasing titers and changing specificities of antinuclear antibodies in patients with chronic liver disease who develop hepatocellular carcinoma. *Cancer*.

[104] Imai H., Ochs R. L., Kiyosawa K., Furuta S., Nakamura R. M., Tan E. M. (1992). Nucleolar antigens and autoantibodies in hepatocellular carcinoma and other malignancies. *The American Journal of Pathology*.

[105] Lu M., Nakamura R. M., Dent E. D. B. (2001). Aberrant expression of fetal RNA-binding protein p62 in liver cancer and liver cirrhosis. *The American Journal of Pathology*.

[106] Kessler S. M., Pokorny J., Zimmer V. (2013). *IGF2* mRNA binding protein p62/IMP2-2 in hepatocellular carcinoma: antiapoptotic action is independent of IGF2/PI3K signaling. *American Journal of Physiology-Gastrointestinal and Liver Physiology*.

[107] Cariani E., Lasserre C., Seurin D. (1988). Differential expression of insulin-like growth factor II mRNA in human primary liver cancers, benign liver tumors, and liver cirrhosis. *Cancer Research*.

[108] Tybl E., Shi F. D., Kessler S. M. (2011). Overexpression of the *IGF2*-mRNA binding protein *p62* in transgenic mice induces a steatotic phenotype. *Journal of Hepatology*.

[109] Su T. S., Liu W. Y., Han S. H. (1989). Transcripts of the insulin-like growth factors I and II in human hepatoma. *Cancer Research*.

[110] Thomas H. (2016). NAFLD: loss of CD4^+^ T cells in HCC. *Nature Reviews. Gastroenterology & Hepatology*.

[111] Stickel F., Hellerbrand C. (2010). Non-alcoholic fatty liver disease as a risk factor for hepatocellular carcinoma: mechanisms and implications. *Gut*.

[112] Liu Y., Meyer C., Muller A. (2011). IL-13 induces connective tissue growth factor in rat hepatic stellate cells via TGF-*β*–independent Smad signaling. *The Journal of Immunology*.

[113] Shimamura T., Fujisawa T., Husain S. R., Kioi M., Nakajima A., Puri R. K. (2008). Novel role of IL-13 in fibrosis induced by nonalcoholic steatohepatitis and its amelioration by IL-13R-directed cytotoxin in a rat model. *The Journal of Immunology*.

[114] Wang W. W., Ang S. F., Kumar R. (2013). Identification of serum monocyte chemoattractant protein-1 and prolactin as potential tumor markers in hepatocellular carcinoma. *PLoS One*.

[115] Wicki A., Mandalà M., Massi D. (2016). Acquired resistance to clinical cancer therapy: a twist in physiological signaling. *Physiological Reviews*.

[116] Vychytilova-Faltejskova P., Radova L., Sachlova M. (2016). Serum-based microRNA signatures in early diagnosis and prognosis prediction of colon cancer. *Carcinogenesis*.

[117] Ballard-Barbash R., Friedenreich C. M., Courneya K. S., Siddiqi S. M., McTiernan A., Alfano C. M. (2012). Physical activity, biomarkers, and disease outcomes in cancer survivors: a systematic review. *Journal of the National Cancer Institute*.

[118] Li M., Gu J. (2005). Changing patterns of colorectal cancer in China over a period of 20 years. *World Journal of Gastroenterology*.

[119] Pisani P., Bray F., Parkin D. M. (2002). Estimates of the world-wide prevalence of cancer for 25 sites in the adult population. *International Journal of Cancer*.

[120] Liu W., Wang P., Li Z. (2009). Evaluation of tumour-associated antigen (TAA) miniarray in immunodiagnosis of colon cancer. *Scandinavian Journal of Immunology*.

[121] Carey L. A., Perou C. M., Livasy C. A. (2006). Race, breast cancer subtypes, and survival in the Carolina Breast Cancer Study. *JAMA*.

[122] Su Y., Qian H., Zhang J., Wang S., Shi P., Peng X. (2005). The diversity expression of p62 in digestive system cancers. *Clinical Immunology*.

[123] Zhang L., Liu Y., Hao S., Woda B. A., Lu D. (2011). IMP2 expression distinguishes endometrioid from serous endometrial adenocarcinomas. *The American Journal of Surgical Pathology*.

[124] Lax S. F., Pizer E. S., Ronnett B. M., Kurman R. J. (1998). Clear cell carcinoma of the endometrium is characterized by a distinctive profile of p53, Ki-67, estrogen, and progesterone receptor expression. *Human Pathology*.

[125] Yemelyanova A., Ji H., Shih I. M., Wang T. L., Wu L. S. F., Ronnett B. M. (2009). Utility of p16 expression for distinction of uterine serous carcinomas from endometrial endometrioid and endocervical adenocarcinomas: immunohistochemical analysis of 201 cases. *The American Journal of Surgical Pathology*.

[126] Batlle E., Clevers H. (2017). Cancer stem cells revisited. *Nature Medicine*.

[127] Fiori M. E., Villanova L., De Maria R. (2017). Cancer stem cells: at the forefront of personalized medicine and immunotherapy. *Current Opinion in Pharmacology*.

[128] Hamilton K. E., Noubissi F. K., Katti P. S. (2013). IMP1 promotes tumor growth, dissemination and a tumor-initiating cell phenotype in colorectal cancer cell xenografts. *Carcinogenesis*.

[129] Chen C. L., Tsukamoto H., Liu J. C. (2013). Reciprocal regulation by TLR4 and TGF-*β* in tumor-initiating stem-like cells. *The Journal of Clinical Investigation*.

[130] Palanichamy J. K., Tran T. M., Howard J. M. (2016). RNA-binding protein IGF2BP3 targeting of oncogenic transcripts promotes hematopoietic progenitor proliferation. *The Journal of Clinical Investigation*.

[131] Gong C., Li Z., Ramanujan K. (2015). A long non-coding RNA, *LncMyoD*, regulates skeletal muscle differentiation by blocking IMP2-mediated mRNA translation. *Developmental Cell*.

[132] Zhou X., Li M., Huang H. (2016). HMGB2 regulates satellite-cell-mediated skeletal muscle regeneration through IGF2BP2. *Journal of Cell Science*.

[133] Mineo M., Ricklefs F., Rooj A. K. (2016). The long non-coding RNA HIF1A-AS2 facilitates the maintenance of mesenchymal glioblastoma stem-like cells in hypoxic niches. *Cell Reports*.

[134] Giovannucci E., Harlan D. M., Archer M. C. (2010). Diabetes and cancer: a consensus report. *CA: a Cancer Journal for Clinicians*.

[135] Shlomai G., Neel B., LeRoith D., Gallagher E. J. (2016). Type 2 diabetes mellitus and cancer: the role of pharmacotherapy. *Journal of Clinical Oncology*.

[136] Voutsadakis I. A. (2017). Obesity and diabetes as prognostic factors in patients with colorectal cancer. *Diabetes & Metabolic Syndrome*.

[137] Scappaticcio L., Maiorino M. I., Bellastella G., Giugliano D., Esposito K. (2017). Insights into the relationships between diabetes, prediabetes, and cancer. *Endocrine*.

[138] Livingstone C., Borai A. (2014). Insulin-like growth factor-II: its role in metabolic and endocrine disease. *Clinical Endocrinology*.

[139] Rose D. P., Haffner S. M., Baillargeon J. (2007). Adiposity, the metabolic syndrome, and breast cancer in African-American and white American women. *Endocrine Reviews*.

[140] Diabetes Genetics Initiative of Broad Institute of Harvard and MIT, Lund University, Novartis Institutes of BioMedical Research (2007). Genome-wide association analysis identifies loci for type 2 diabetes and triglyceride levels. *Science*.

[141] Grarup N., Rose C. S., Andersson E. A. (2007). Studies of association of variants near the *HHEX*, *CDKN2A/B*, and *IGF2BP2* genes with type 2 diabetes and impaired insulin release in 10,705 Danish subjects: validation and extension of genome-wide association studies. *Diabetes*.

[142] Zeggini E., Weedon M. N., Lindgren C. M. (2007). Replication of genome-wide association signals in UK samples reveals risk loci for type 2 diabetes. *Science*.

[143] Omori S., Tanaka Y., Takahashi A. (2008). Association of *CDKAL1, IGF2BP2, CDKN2A/B, HHEX, SLC30A8*, and *KCNJ11* with susceptibility to type 2 diabetes in a Japanese population. *Diabetes*.

[144] Sainz J., Rudolph A., Hoffmeister M. (2012). Effect of type 2 diabetes predisposing genetic variants on colorectal cancer risk. *The Journal of Clinical Endocrinology & Metabolism*.

[145] Meyer T. E., Boerwinkle E., Morrison A. C. (2010). Diabetes genes and prostate cancer in the Atherosclerosis Risk in Communities study. *Cancer Epidemiology Biomarkers & Prevention*.

[146] Conway A. E., Van Nostrand E. L., Pratt G. A. (2016). Enhanced CLIP uncovers IMP protein-RNA targets in human pluripotent stem cells important for cell adhesion and survival. *Cell Reports*.

[147] Li Z., Zhang Y., Ramanujan K., Ma Y., Kirsch D. G., Glass D. J. (2013). Oncogenic NRAS, required for pathogenesis of embryonic rhabdomyosarcoma, relies upon the HMGA2-IGF2BP2 pathway. *Cancer Research*.

[148] Tan E. M., Zhang J. (2008). Autoantibodies to tumor-associated antigens: reporters from the immune system. *Immunological Reviews*.

